# Discovery of a Novel Accessory Structure of the Pitviper Infrared Receptor Organ (Serpentes: Viperidae)

**DOI:** 10.1371/journal.pone.0090622

**Published:** 2014-03-04

**Authors:** Wilmar Bolívar-G, Marta M. Antoniazzi, Taran Grant, Carlos Jared

**Affiliations:** 1 Departamento de Biología, Programa de Posgrado y Grupo de Investigación en Ecología Animal, Universidad del Valle, Cali, Valle del Cauca, Colombia; 2 Laboratório de Biologia Celular, Instituto Butantan, São Paulo, São Paulo, Brazil; 3 Departamento de Zoologia, Instituto de Biociências, Universidade de São Paulo, São Paulo, São Paulo, Brazil; Universität Bielefeld, Germany

## Abstract

The facial pits of rattlesnakes, copperheads, lanceheads, bushmasters and other American and Asian pitvipers (Crotalinae) are highly innervated and densely vascularized infrared (IR) receptor organs. For over a century, studies have focused on a small sample of model species from North America and Asia. Based on an expanded survey of Central and South American crotalines, we report a conspicuous accessory structure composed of well-defined papillae that project from the anterior orbital adnexa. The papillae are continuous with the inner chamber of the IR receptor organ and our histological and ultrastructural data suggest that they possess a well-developed nervous network and extensive vascularization; however, they lack the characteristic IR-sensitive terminal nerve masses found in the IR-receptive pit membrane. The function of the IR receptor organ papillae is unknown.

## Introduction

**Figure 1:**
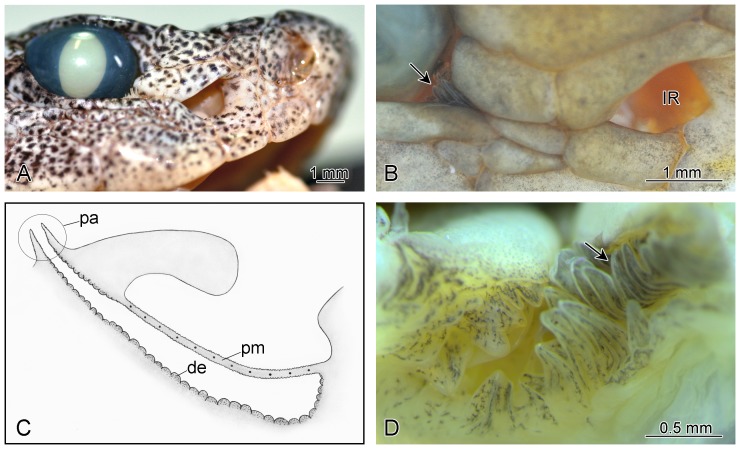
Pitviper infrared receptor organ. (A) Lateral view of *Bothriopsis taeniata* head. (B) High magnification showing infrared (IR) receptor organ pit opening and papillae (arrow) emerging from the anterior corner of the orbit in *Bothrops asper*. (C) Schematic representation of the IR receptor organ showing the location of the papillae (pa) in relation to pit membrane (pm) and domed epithelium (de) of the bottom of the inner chamber (after [2], [7]). (D) View of the anterior orbital adnexa showing the IR receptor organ pore encircled by papillae in *Bothrops asper*. The arrow points to the more elongate papillae in the anterior corner of the orbit.

Rattlesnakes, copperheads, lanceheads, bushmasters and other American and Asian pitvipers (Crotalinae) are uniquely characterized by possessing a deep facial pit in the loreal region between the eye and naris (Figures 1A, B). Since Noble and Schmidt [1] demonstrated the heat sensing function of crotaline facial pits, the mechanism of infrared (IR) detection has been elucidated in remarkable detail, resulting in an intricate understanding of the mechanism of IR detection and its functional and evolutionary significance [2]–[4]. Internally, the facial pit is composed of outer and inner chambers separated by a thin IR-receptive membrane [5] (Figure 1C). The air-filled inner chamber extends posteriad via a duct that opens into the anterior orbital adnexa [5], [6] to equalize atmospheric pressure inside the chamber [2]. The pit is innervated by the ophthalmic and maxillary ganglia of the trigeminal nerve, and the pit membrane is imbedded with unique, IR-sensitive terminal nerve masses (TNMs) [7]–[9]. Detection of infrared radiation occurs through thermosensitive transient receptor potential TRPA1 channels on sensory nerve fibers that innervate the pit membrane [4]. In addition to being densely innervated, the pit membrane is vascularized by a dense capillary bed [5] to meet high energy and oxygen demands and enable rapid heat exchange for receptors to immediately return to background temperatures [10], [11]. The external surface of the pit membrane contains pores or micropits that allow long wavelengths to pass and stimulate receptors while filtering short, visible-spectrum wavelengths [12]. The surface of the bottom of the inner chamber is arranged into domes of variable sizes that help prevent infrared rays from reflecting back onto the pit membrane [2].

Previous studies of IR receptor organ structure and function focused on only a few species of four genera (*Gloydius* and *Trimeresurus* from Asia, *Agkistrodon* and *Crotalus* from North America), with little or nothing known about IR receptor organs of the remaining 200 species of crotalines. While conducting an expanded survey of the IR receptor organ in Central and South American crotalines, we discovered a new accessory structure that is extensively innervated, highly vascularized, and continuous with the IR receptor organ but that lacks the IR-sensitive TNMs present in the pit membrane.

## Materials and Methods

For convenience, we follow the generic taxonomy of Fenwick et al. [13], which recognizes five genera for bothropoids (for an alternative taxonomy see [14]). We examined *Bothriechis schlegelii*, *Bothropoides jararaca*, *Bothrops asper*, *Bothrops brazili*, and *Crotalus durissus* with light and electron microscopy. For light microscopy (LM), fixed samples were also dehydrated in ethanol and embedded in glycol methacrylate (Leica). Sections (3–4 µm) were cut in a Leica RM 2255 microtome and stained with toluidine blue-fuchsin [15]. Images were obtained in an Olympus BX51 microscope using an Olympus Q-Color 5 digital camera and Image Pro Express 5.0 (Media Cybernetics) software. For electron microscopy, IR receptor organs were extracted and fixed in 2.5% glutaraldehyde in 0.1 M phosphate buffer. For scanning electron microscopy (SEM), the samples were dehydrated in an increasing series of ethanol, dried in a critical point dryer using CO_2_ as an intermediate medium, and coated with gold in a sputtering device. Samples were examined in an FEI Quanta 250 scanning electron microscope, operating at 10–12.5 kV. For transmission electron microscopy (TEM), the samples were post-fixed in 1% osmium tetroxide, dehydrated and embedded in epoxy resin. Ultrathin sections were cut in a Leica UC7 ultramicrotome, contrasted in 2% uranyl acetate and lead citrate and examined in a LEO 906E electron microscope operating at 80 kV.

Dissecting microscopes were used for gross examination of duct openings of the following specimens (MZUSP: Museu de Zoologia da Universidade de São Paulo; UV-C: Colección de Anfibios y Reptiles de la Universidad del Valle; CD: Colección de Vertebrados de Docencia de la Universidad del Valle): *Atropoides nummifer* (MZUSP 2030, 8231); *Agkistrodon piscivorus* (MZUSP 3020–3022, 9348); *Bothriechis schlegelii* (UV-C 6665, 5433; CD 334–338, 340, 343–345, 347, 349–350, 743–744, 2433, 2493, 2496, 2499, 2500); *Bothriopsis bilineata* (MZUSP 1436, 3722, 4398, 4456, 8253, 10371–10372, 13248, 15173, 19033); *Bothriopsis taeniata* (MZUSP 11234–35, 11253, 11575); *Bothrocophias hyoprora* (MZUSP 5293); *Bothropoides alcatraz* (MZUSP 1453); *Bothropoides jararaca* (MZUSP 5252); *Bothrops asper* (UV-C 10755, 10762, 10764, 10804, 11328, 13016, 13310, 13825, 15266, 15273, 15276, 15593, 15630, 16268; CD 2434, 2501); *Bothrops brazili* (MZUSP 4260, 4895, 10534, 11719, 18128, 19295–19298); *Bothrops jararacussu* (MZUSP 14923); *Crotalus durissus* (MZUSP 7308; CD 1813, 2344–2351, 2492, 2494–2495, 2497–2498); *Lachesis muta* (MZUSP 5330, 5719); *Ovophis okinavensis* (MZUSP 2278, 7701); *Porthidium lansbergi* (MZUSP 7733, 7769, 7795); *P. nasutum* (MZUSP 7480; CD 322, 1665); *Rhinocerophis alternatus* (MZUSP 1458); *R. cotiara* (MZUSP 2504).

### Ethics statement

Permission was obtained from all the relevant museums/institutions to access the collections and specimens were either loaned or examined at the museum/institution.

## Results

We examined the gross morphology of the IR receptor organs of 18 species of pitvipers representing the phylogenetic diversity of Neotropical lineages and one Asian genus. In all examined species of the Neotropical genera *Bothriopsis*, *Bothrocophias*, *Bothropoides*, *Bothrops*, *Lachesis*, *Porthidum*, and *Rhinocerophis* the orbital opening of the inner chamber duct lies deep in the orbit and is encircled by well defined cuneiform papillae that vary in length from greatly enlarged folds to elongate, externally visible projections emerging from the anterior corner of the orbit (Figure 1). In contrast, the orbital aperture of the inner chamber duct of *Agkistrodon piscivorus*, *Atropoides nummifer*, *Bothriechis schlegelii*, *Crotalus durissus*, and *Ovophis okinavensis* is a simple preocular opening in the superficial orbital adnexa, immediately beneath the preocular scales, which is consistent with previous observations [2].

**Figure 2:**
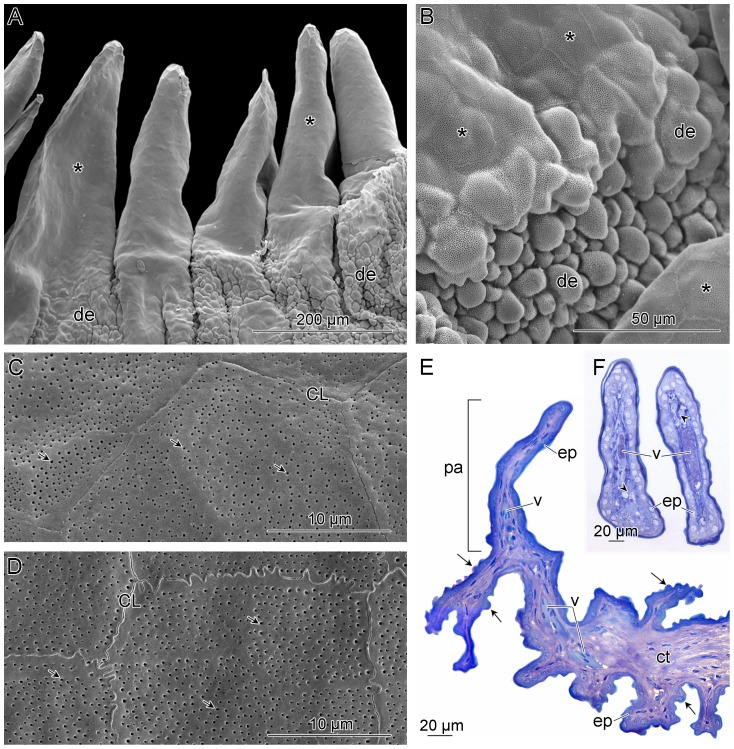
Microscopy of the pitviper infrared receptor organ. (A–D) Scanning electron micrographs of the papillae of *Bothropoides jararaca*. (A) General view and (B) higher magnification of the base of the papillae, focusing on the transition from the flat epithelium (*) to the domed epithelium (de) that lines the duct and bottom of the inner chamber. (C, D) High magnification of the surface of the (C) papilla and (D) pit membrane. Note the cell limits (CL) and micropits (arrows). (E, F) Light micrographs of *Bothrops asper*. (E) Longitudinal section of one papilla (pa) and part of the inner chamber showing epithelial domes (arrows). (F) Transverse section of two papillae. Note the epithelial cells (ep) and large blood vessels (v) running within the internal connective tissue (ct).

LM and SEM revealed that the surface of the papillae is flat (Figures 2A, E, F), with the epithelium at the papillary base gradually folding to form the same domed surface as the adjacent duct and pit fundus (Figure 2B). The epithelial surface of the papillae (Figure 2C) is nearly indistinguishable from that of the IR-receptive pit membrane (Figure 2D). In both, the epithelial cells are polygonal and possess the same distinct, evenly distributed pores or micropits separated by broad, smooth lines.

**Figure 3:**
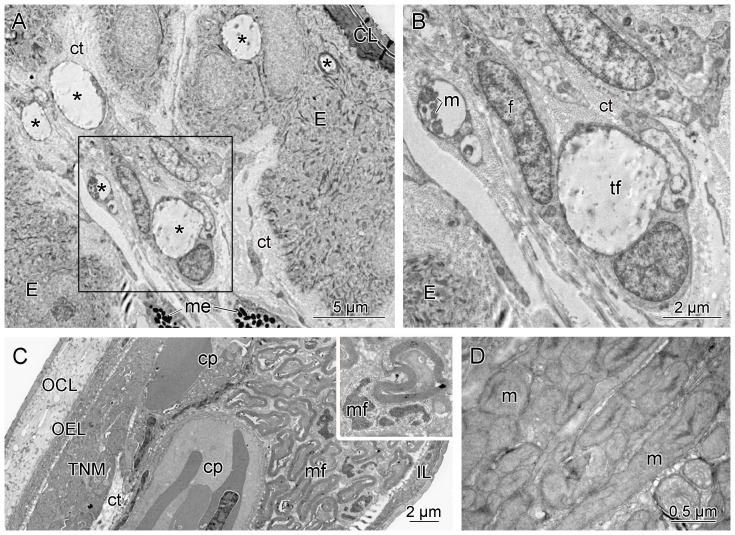
Transmission electron microscopy of the infrared receptor organ papillae and the pit membrane of *Bothrops asper*. (A) Transverse section of a papilla showing the epidermis (E) covered by a cornified layer (CL) and the internal connective tissue (ct) with many free terminal nerve fibers (*), some of which penetrate the epidermis. Melanophores (me) are also present. (B) Higher magnification of the rectangle in (A) showing the non-myelinated terminal fibers (tf), fibroblasts (f), and arrangement of mitochondria (m). (C) Infrared receptor organ pit membrane with typical outer cornified layer (OCL), outer epithelial layer (OEL), terminal nerve masses (TNM), capillaries (cp), myelinated fibers (mf) (in higher magnification in the insert), and inner layers (IL). (D) Detail of a TNM showing densely packed mitochondria (m).

LM and TEM of the papillae of *Bothrops asper* showed that the papillae are covered by an epithelium composed of two layers of cells and a cornified superficial layer (Figures 2E, 3A). The interior of the papillae is composed of connective tissue through which run blood vessels and capillaries (Figure 2E) and an extensive nervous network. Non-myelinated free terminal fibers are distributed throughout the entire papilla, penetrating the epithelial cells and almost reaching the cornified layer (Figures 3A, B). Melanophores are common within the connective tissue (Figure 3A). The IR-receptive pit membrane of *B. asper* shows the same general pattern described previously [2], [9], with a thick outer cornified layer, conspicuous TNMs distributed below the outer epithelial layer, and many myelinated nerve fibers especially concentrated around blood vessels (Figures 3C–D). Although a high number of free terminal fibers occur throughout the IR receptor organ papillae, similarly organized nervous structures were not observed in the papillae.

## Discussion

The function of the IR receptor organ papillae is unknown. In general, papillae and papilla-like structures increase surface area to fulfill a variety of sensory and physiological functions. Despite the considerable increase in epithelial surface area, there is no evidence that papillary tissues or cells are used for absorption. Likewise, there is no indication of a secretory function associated with either isolated cells or clusters of cells or of any storage of secretory compounds. Primarily chemoreceptive and mechanoreceptive/tactile functions are possible but seem unlikely given the location of the papillae in the anterior corner of the orbit. Given their position around the orbital opening of the inner chamber duct, the papillae could serve as a physical barrier to prevent debris or ectoparasites from entering the inner chamber and interfering with IR detection while still allowing inner chamber pressure to equalize. However, this hypothesis is inconsistent with their extensive vascularization and and thin epithelium.

The fact that the papillae are projections of the IR receptor organ into the environment from the orbit suggests they might be related somehow to IR-reception, possibly functioning as a second IR detector that provides an independent reference point to complement the information obtained by the pit membrane. The extensive vascularization of the papillae, which is necessary for energy and oxygen demands and rapid heat exchange in the IR-receptive pit membrane [10], [11], is also consistent with the latter hypothesis. However, IR reception in the pit membrane occurs in the IR-sensitive TNMs [2], which are absent in the papillae. An alternative hypothesis is that the highly vascularized papillae function in heat exchange, although quantitative comparisons with reference structures are necessary to determine the significance of the observed vascularization.

Clearly, the discovery of this accessory structure of the pitviper IR receptor organ raises more questions than can be answered presently. If the IR receptor organ papillae are directly involved in IR reception, how do they accomplish this function without IR-sensitive TNMs? And if they are not directly involved in IR reception, then what function do they perform? Previous studies of IR receptor organ structure and function focused on only a few species of the Asian genera *Gloydius* and *Trimeresurus* and the North American genera *Agkistrodon* and *Crotalus*—none of which possess papillae; does IR reception differ between species that possess and lack IR receptor organ papillae? Despite recent efforts to elucidate viperid phylogeny [13]–[14], [16]–[18], relationships are not yet clear enough to determine precisely the evolutionary history of the IR receptor organ papillae. Nevertheless, IR receptor organ papillae occur in almost all South American lineages of pitvipers and, therefore, characterize a large proportion of the diversity of Crotalinae. What role did this accessory structure play in the evolutionary radiation of the Neotropical pitvipers, and what are the ecological correlates of the presence and absence of the papillae and variations in papillary size and shape? To answer these and many more questions, broad, phylogenetically informed surveys of pitviper diversity must be combined with the kinds of intensive studies, including electrophysiological and behavioral studies, of model organisms that have characterized the past century of research on IR reception.
